# A novel calibration strategy based on internal standard–spiked gelatine for quantitative bio-imaging by LA-ICP-MS: application to renal localization and quantification of uranium

**DOI:** 10.1007/s00216-020-02561-4

**Published:** 2020-03-20

**Authors:** Nagore Grijalba, Alexandre Legrand, Valerie Holler, Céline Bouvier-Capely

**Affiliations:** Institut de Radioprotection et de Sûreté Nucléaire, PSE-SANTE/SESANE/LRSI, BP17, 92262 Fontenay-aux-Roses Cedex, France

**Keywords:** Uranium, Kidney, Bio-imaging, Quantification, Internal standardization, LA-ICP-MS

## Abstract

Mass spectrometry imaging (MSI) using laser ablation inductively coupled plasma mass spectrometry (LA-ICP-MS) has been employed for the elemental bio-distribution and quantification of uranium (U) in histological tissue sections of rodent kidneys. Kidneys were immediately immersed into 4% paraformaldehyde (PFA) solution for 24 h, Tissue-Tek O.C.T. Compound embedded and stored at − 80 °C until cutting in a cryostat, and mounted in gel-covered glass slides. In order to assure complete ablation of sample, sample preparation and laser conditions were carefully optimized. In this work, a new analytical methodology is presented for performing quantitative laser ablation analyses based on internal standard (thulium, Tm)–spiked gelatine (10% m/v) for correction of matrix effects, lack of tissue homogeneity, and instrumental drift. In parallel, matrix-matched laboratory standards, dosed at different concentrations of U, were prepared from a pool of rat kidneys. The quantitative images of cryo-sections revealed heterogeneous distribution of uranium within the renal tissue, because the cortical concentration was up to 120-fold higher than the medullary concentration.

**Figure:**
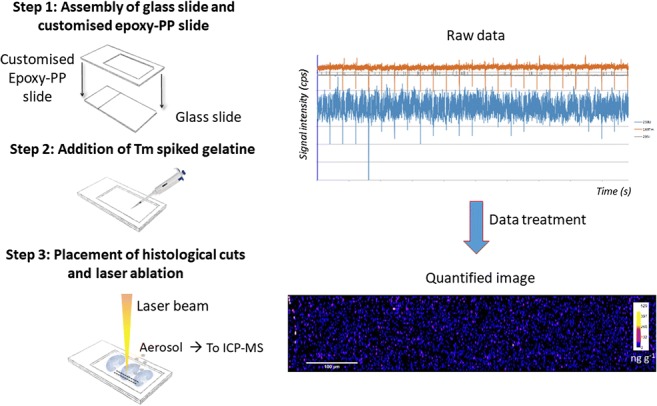
Graphical abstract

Graphical abstract

## Introduction

The quantitative analysis of trace metals in different organs or cellular structures is a topic of emerging interest for toxicological risk assessment. Uranium is a heavy metal that possesses radioactive properties. This natural radioelement (UNat) is ubiquitously present in the earth’s crust (soil, water, and air) which implies the exposition of the general public population by ingestion and inhalation. Whatever the route of exposure, uranium is distributed all over the tissues by the bloodstream and it is preferentially accumulated in kidneys and bones [1, 2].

The kidney is recognized as a major site for uranium accumulation which may induce renal toxicity. This toxic effect, exerted both by chemical and radiological actions, is characterized by reduced glomerular filtration [3–5]. In addition, its tissue distribution is heterogeneous, mostly accumulating and producing pathological lesions in the proximal tubule located in the cortical zone (uranium concentration 100-fold above mean renal concentration) [6–8]. Chronic exposure (occupational exposure) to uranium could be related to its bioaccumulation in the kidney and associated with renal dysfunction and an increased risk of cancer mortality and kidney failure [9–11].

The internal doses are used for the evaluation of health risk after internal contamination by radionuclides, which are based on their biokinetics and dosimetric models [12] . The International Commission on Radiological Protection (ICRP) is in charge of developing these models and describes the radionuclides behavior after their incorporation into the organism (ingestion and/or inhalation), transfer to the blood, and the absorption of the energy resulting from their nuclear transformations in the target organ [13].

Paquet et al. showed that the uranium accumulation after chronic exposure is overestimated by the use of a model designed for acute exposure and suggested the need of specific models for the calculation of dose resulting from chronic intake of radionuclides [14, 15]. Due to uranium heterogeneous distribution within the tissue, the quantification at the tissue level appears to be of significant interest. The precise location and accurate quantification of uranium in rodents’ kidneys would facilitate trustworthy information for reconstructing a more realistic dosimetry model for a better estimation of health risk that could be related to renal dysfunction or cancer occurrence in workers exposed to uranium. To date, few quantitative studies have been carried out employing high-energy synchrotron radiation X-ray fluorescence (SR-XRF) analysis and X-ray absorption fine structure (XAFS) [16, 17]. Unfortunately, the limited access to these analytical techniques limits considerably its daily use for routine analysis [18]. To the author’s knowledge, a single work has been published recently for the semi-quantitative analysis of uranium by LA-ICP-MS in mice kidneys [19] due to the lack of an appropriate internal standard. In this work, LA-ICP-MS has been employed for mapping and quantifying uranium in histological tissue sections of rat kidney.

Laser ablation inductively coupled plasma mass spectrometry (LA-ICP-MS) has evolved as an efficient analytical technique for in situ quantitative analysis of solid samples with micrometer-scale spatial resolution and low limits of detection [20]. Especially, elemental bio-imaging has become more and more popular in the last decade for in situ localization and quantification of trace metals in a wide range of matrices, allowing the reconstruction of isotope-specific maps [21]. However, the progress in biological or medical science application is still determined by the availability of calibration standards and appropriate internal standards (IS) to compensate signal variation during laser beam-sample interaction, transportation of the aerosol, and instrumental drifts during analysis [22, 23]. The internal standard is generally an element that is spiked at a known concentration into the matrix or a major element that is already present in the matrix, whose concentration has been previously quantified by other analytical methods [24]. It must be highlighted that if the concentration of the internal standard is not known, it would not be possible to determine the concentration of the analyte of interest with accuracy and precision [25]. The preparation of matrix-matched standards from material with the same matrix as the sample is a widely used approach for quantification purposes [26]. In this work, in-house solid matrix–matched standards were prepared from uranium-spiked kidney homogenate. Nevertheless, further calibration strategies are found in the literature including the solution-based calibration [27], fabrication xerogel solid calibration standards which are more suitable for the calibration of glasses and silicate matrices [28], the synthesis of metal-spiked polymer films [29], the use of dried droplets or dried matrix spots (DMS) deposited on different substrates [30–34], and the spiking and sectioning of gelatine droplets, whose use has increased noticeably in recent years for the quantitative analysis of biological tissues [35–41]. For internal standardization, three different approaches are mainly described in the literature for the quantitative elemental imaging of biological samples: (i) the simultaneous nebulization of a standard solution [42], (ii) the use of an element naturally occurring in the sample [43–45], and (iii) the addition of an internal standard–spiked layer between the sample and support or on the sample [46–51]. In this study, a thulium (IS)-doped gelatine, located between the support and the tissue, was prepared in order to incorporate a more appropriate internal standard than ^13^C, as its feasibility for quantitative LA-ICP-MS imaging has been queried recently [52–54]. Thulium (169, monoisotopic) was chosen as IS due to its high atomic mass and first ionization potential (FIP) which is close to that of uranium, 6.184 eV and 6.050 eV, respectively. Gelatine gel was chosen the IS substrate for mimicking the texture of biological tissues, ease of handling, and fast preparation, and the number of analytes and their concentration range can be adapted [37].

## Materials and methods

### Standards, reagents, and samples

The chemicals and reagents were of analytical grade. High-purity standard solutions of uranium and thulium (1000 μg mL^−1^) were purchased from SPEX CertiPrep (Stanmore, UK). Acid digestion of biological tissue was performed using nitric acid (67–69% HNO_3_ for trace metal analysis, VWR International, Leuven, Belgium) and hydrogen peroxide (30% H_2_O_2_, Merck Schuchardt, Hohenbrunn, Germany). Enriched uranium solution for isotope dilution (ID)-ICP-MS analysis was provided by IRSN (U^236^ 49.51% isotopic abundance, 616 mBq). Type A gelatine (porcine skin, 225 bloom) was obtained from MP Biomedicals (Illkirch, France). All solutions were prepared using distilled deionized water (18.2 MΩ cm^−1^) obtained from a Milli-Q water purification system (Millipore, Bedford, MA, USA). Thermo Scientific SuperFrost Plus Adhesion glass slides were used as support for the gelatine (VWR). In addition, in order to enclose the gelatine, customized polymeric slides (inner area of 45.1 × 16 × 1 mm) were purchased from SymaLab (Pau, France).

### Uranium exposure and biological sample preparation

The samples used in this study were ceded by the Laboratory of Radiotoxicology (IRSN PSE-SANTE/SESANE/LRTOX). These samples were employed for the work of Poisson et al. and they are nowadays a part of IRSN tissue bank. As a brief summary of sample preparation employed in this work, Sprague-Dawley rats were exposed to varying concentrations of uranium in their drinking water for a period of 9 months. A control group received non-contaminated water. The remaining experimental groups received a constant supply of water contaminated with uranyl nitrate hexahydrate, UO_2_ (NO_3_)_2_·6 H_2_O (^238^U, 99.74%; ^235^U, 0.26%; ^234^U, 0.001%). The uranium concentrations ranged from environmental concentrations (1 mg L^−1^) to levels described as nephrotoxic (120 and 600 mg L^−1^). Each group contains 12 animals. After the corresponding contamination periods, the rats were euthanized and both kidneys were collected. Half the left kidney was placed in PFA 4% for 24 h and Tissue-Tek OCT embedded, which was employed for LA-ICP-MS analysis. The other half was acid-digested and the total amount of uranium was quantified by ICP-MS. Detailed information about the experimental setup and authorization involving animals are given in the study published by Poisson et al. [55]. Sample preparation of biological samples is a crucial step due to their uniqueness and reduced availability. There are well-known histology sample preparation strategies for tissue preservation but they could lead to metal content modification, due to leaching of the metals from tissue to different fixing solutions [56]. Bonta et al. and Hare et al. demonstrated that fixed samples are completely unsuitable for the LA-ICP-MS analysis of group 1 and 2 metals while the results obtained for transition metals are comparable with those of snap-frozen samples but any mention to actinides is referenced [57, 58]. Nevertheless, biological samples are often precious samples and are shared to perform different types of analysis and a compromise between sample preparation strategies must be reached to satisfy the requirements of each analysis. In this study, kidneys were kept in PFA (24 h) and OCT-embedded prior to analysis. The authors are aware that this sample preparation might distort the initial metal concentrations but it poorly alters its distribution [59]. However, the experimental procedure was rigorously applied to minimize the experimental bias. Three samples were selected for the current MSI analysis whose total uranium concentrations are the following: sample 1 (control sample, 4 ng g^−1^ U), sample 2 (contaminated sample, 3300 ng g^−1^ U), and sample 3 (contaminated sample, 6700 ng g^−1^ U). Longitudinal sections, 16-μm thickness, were cut in a Thermo Scientific Microm HM 500 cryostat at − 20 °C, deposited in gelatine-covered glass slides, and preserved at 4 °C until LA-ICP-MS bio-imaging analysis.

### Preparation of internal standard–spiked gelatine

2.5%, 5%, 10%, 20%, and 30% (m/v) gelatines were prepared for the optimization of internal standard–spiked gelatine, all of them containing 35 μg g^−1^ of thulium. A minimum volume of standard (25 μL) was added since acid medium could trigger gelatine degradation and thus affect the homogeneous distribution of the internal standard. The solution was mixed with soft magnetic agitation at 80 °C avoiding bubble formation. After, 0.5 mL of gelatine was carefully deposited on top of the glass slides. Slides were after left drying at room temperature (1 h, covered) and then kept at 4 °C until their use. Internal standard concentration in gelatine was verified by ICP-MS analysis after microwave-assisted acid digestion of a gelatine aliquot (quantification carried out by external calibration).

### Preparation of matrix-matched standards

Matrix-matched standards were synthesized for the calibration and quantification of images obtained by LA-ICP-MS. For this purpose, a homogenate of analogous tissue was prepared and spiked with known amounts of uranium increasing concentrations. The animals were housed in the IRSN animal facilities accredited by the French Ministry of Agriculture for performing experiments on live rodents. Animal experiments were performed in compliance with French and European regulations on the protection of animals used for scientific purposes (EC Directive 2010/63/EU and French Decret 2013–118). All experiments were approved by the Ethics Committee #81 under the reference P19-25 (internal project number; no official authorization required as animals were euthanized for tissue sampling). Fourteen rat kidneys, harvested in our animal facility, were homogenized using a manual mincer resulting in ~ 25 g of homogenate. The homogenate was then divided into ten aliquots (~ 2.3 g each). Six aliquots were used for matrix-matching standards, three aliquots were used for quality control samples (low QC, middle QC, and high QC), and the latest was kept for blank. Indeed, according to the Guideline on Bioanalytical Method Validation, a minimum of six calibration concentrations should be used, in addition to the blank sample and a minimum of three QC samples representing the entire range of the standard curve [60]. The added uranium concentrations in matrix-matched standards were selected in order to cover the entire range of expected concentrations, from 5 to 15,000 ng g^−1^ according to the acid digestion and subsequent analysis by ICP-MS of the whole organ [55]. All standards were then carefully vortexed for homogenization, filled into 0.5 cm^3^ histology moulds, and frozen at − 20 °C. Uranium elemental concentrations in kidney homogenates were validated by ID-ICP-MS analysis after microwave-assisted acid digestion (ETHOS One, Millestone Srl, Sorisole, Italy). For this purpose, an aliquot of 100 mg of each standard (*n* = 3) was digested in 3 mL of 67–69% HNO_3_ and 1.5 mL 30% H_2_O_2_. Verified uranium concentrations on standards are shown in Table 1. Finally, standards were cut on a cryostat at − 20 °C in 16-μm sections and mounted onto gelatine-covered glass slides in the same way as the samples.

**Table 1:** Verified concentrations of matrix-matched standards by ID-ICP-MS. Results are expressed as mean value ± standard deviation (*n* = 9)

Standard (Std)/quality control (QC)	Uranium concentration (ng g^−1^)
QC 1	20 ± 1
Std 1	59 ± 12
Std 2	107 ± 11
Std 3	505 ± 71
QC 2	1042 ± 92
Std 4	6824 ± 649
QC 3	9384 ± 1792
Std 5	14,483 ± 2352
Std 6	16,218 ± 402

### LA-ICP-MS instrumental conditions

For laser ablation, a nanosecond Analyte Excite excimer laser ablation system (193 nm) was used (Teledyne CETAC Technologies, Omaha, NE, USA). The laser ablation system is equipped with a standard HelEx laser ablation cell. The laser was operated with a fluence of 6.75 J/cm^2^. The ablated material was transported through a 2-m-long polyurethane tube (i.d. 4 mm) by a helium gas stream (500 mL min^−1^) to the ICP-MS. For ICP-MS measurements, an ICAP-Q (Thermo Scientific, Bremen, Germany) was employed working in wet plasma conditions using a two-inlet nebulization chamber that mixed the dry aerosol together with a nebulized aerosol (1 μg L^−1^ Bi, in 2% HNO_3_) via a pneumatic nebulizer. The LA-ICP-MS coupling was tuned on a daily basis in terms of plasma robustness (^238^U/^232^Th = 1.00 ± 0.05), sensitivity (> 200.000 cps U), and background intensity (< 100 cps U) using SRM NIST 612 glass standard. The optimized experimental conditions are summarized in Table 2.

**Table 2:** Operating conditions of LA-ICP-MS system

Laser ablation system	CETAC Analyte Excite
Wavelength (ArF excimer)	193 nm
Repetition rate	20 Hz
Spot diameter	35 μm
Scan speed	35 μm s^−1^
Fluence	6.75 J cm^2^
Carrier gas (Ar)	0.5 L min^−1^
Wet plasma conditions	1 ppb Bi, 2% HNO_3_, 0.4 rpm
Ablation mode	Single line scan
ICP-MS	ICAP-Q
RF power	1350 W
Nebulizer gas (Ar)	0.65 L min^−1^
Auxiliary gas (Ar)	0.8 L min^−1^
Detector	Dual mode
Dwell time	10 ms
Signal acquisition	Time-resolved analysis (TRA)
Isotopes	^29^Si, ^169^Tm, ^235^U, ^238^U, and ^209^Bi

Standards are placed together with the samples in the ablation cell and they are measured before and after the analysis of the sample to compensate for instrumental drift. Bio-imaging measurements were performed by ablating an area line by line scan ablation using a spot diameter of 35 μm (square mask) and 35-μm interlinear distance. In all the experiments, data was recorded for 20 s before the laser was fired to establish a baseline level (gas blank signal). After the laser was turned off, the signal was recorded for an additional 20 s to confirm that ICP-MS signal returned to its blank values. Data preprocessing consisted of peak-area integration and background subtraction. The baseline was estimated by the average signal measured during the 20 s prior to the ablation and then subtracted point by point from the signals measured for each isotope in the ablation peak. The uranium ICP-MS signal was then normalized relative to the ICP-MS signal of the internal standard (Tm). Data treatment and 2D data visualization of elemental distribution in renal tissue sections were created using a home-made software developed by Dr. Christophe Pécheyran (CNRS, UPPA-IPREM, Pau, France) and ImageJ.

## Results

### Evaluation of internal standard–spiked gelatine

**Fig. 1:**
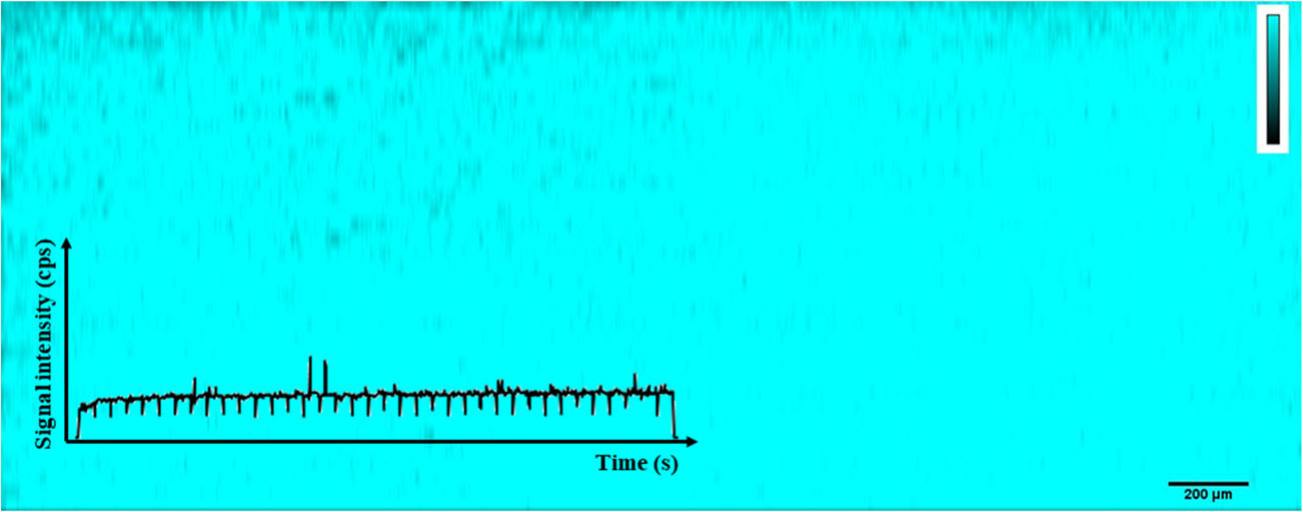
Distribution of Tm in 10% gelatine (ablation area of 3.3 × 1.3 mm) and the corresponding raw data

Gelatine concentrations of 20% and 30% (m/v) were directly discarded due to their high viscosity and the formation of bubbles in the gel film. Gelatine concentrations of 2.5%, 5%, and 10% were spiked with 100 ng g^−1^ of thulium (^169^Tm, FIP = 6.2 eV) and thallium (^205^Tl, FIP = 6.1 eV) and were analyzed by LA-ICP-MS to study element homogeneity. For this purpose, an area of 0.5 × 0.5 mm^2^ was ablated in the upper, middle, and lower part of the slide (*n = 9*), and the Tm/Tl ratio was calculated to evaluate the distribution of both elements. The Tm/Tl ratio was found to be much higher than 1 for the gelatine concentrations of 2.5% (Tm/Tl = 7.1 ± 1.6, RSD = 20%) and 5% (Tm/Tl = 3.5 ± 0.6, RSD = 20%), while the ratio was 1.6 ± 0.08 (RSD = 5%). Additionally, the central parts and edges of the gelatine films (*n = 2*) were cut and analyzed by ICP-MS separately, confirming a homogeneous distribution of both elements (data not shown). These findings come into agreement with the study published by Šala et al. [37] in which they concluded that (1) lower initial gelatine concentrations affect negatively 3D element homogeneity due to a fast setting and drying process leading to the “coffee-stain” effect (higher concentration at the edges) and (2) gelatines with the most homogenous element distributions are prepared from 10% m/v gelatine solutions. Therefore, based on our experimental data by comparing different gelatine percentages and Šala et al.’s work, gelatine concentration was kept at 10% m/v proven as being the most suitable concentration for LA-ICP-MS bio-imaging experiments. Figure 1 shows the homogeneous distribution of Tm in an ablation area of 3.3 × 1.3 mm on Tm-spiked gelatine and the corresponding raw data. Nevertheless, the gelatine lifetime is determined by its biological origin and its high water content, which is an appropriate medium for fungal growth. Gelatine-coated slides must be stored in a refrigerator between 4 and 6 °C and protected in a plastic histological slide folder, and its immediate use is recommended (1 week). Once the histological cut is deposited on the surface of the gelatine, it is preferred to perform LA-ICP-MS measurements as soon as possible.

### Evaluation of matrix-matched standards and calibration curve

**Fig. 2:**
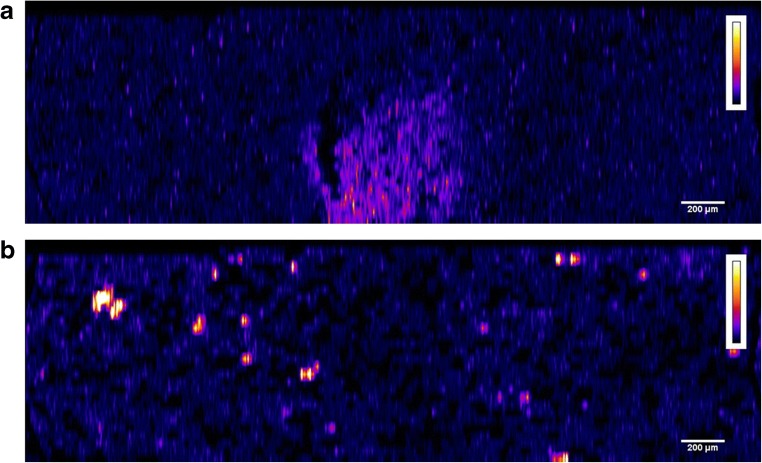
2D images of uranium distribution in matrix-matched standard 2 (a) and matrix-matched standard 5 (b)

Quality control (QC) samples were used to monitor the performance of the method and to assess the integrity and validity of the results of the unknown samples. Five replicates per concentration level were done, obtaining a linear calibration curve with a correlation coefficient of 0.993. The precision was evaluated based on the percent relative standard deviation for the averaged values, ranging from 5 to 25%. In fact, it was found that the micro-distribution of uranium within the standards was not as homogeneous as expected. Figure 2 shows the uranium distribution in matrix-matched standard 2 (a) and matrix-matched standard 5 (b). This fact could be explained by the heterogeneity of kidney homogenates. Indeed, the homogenization procedure applied in this study (manual grinding machine) seems not to be the most adequate method for kidney homogenization due to the fibrous structure of the organ itself. Therefore, it would be desirable to use a more performant homogenization procedure (automated ultrasound homogenization, for example). Otherwise, it could be recommended to increase the ablated area of standards to average the uranium concentration in detriment of the analysis time. Then, QC samples were ablated in triplicate (0.5 × 0.5 mm^2^ area). The calculated concentrations of the QC samples (obtained by LA-ICP-MS) were found to be within the ± 20% of the nominal value (concentrations determined by ID-ICP-MS), validating the proposed quantification method based on thulium-spiked gelatine (Table 3).

**Table 3:** Uranium concentrations of QC samples determined by LA-ICP-MS and comparison with the expected concentrations obtained by ID-ICP-MS. Results are expressed as mean value ± standard deviation (*n* = 3)

	Obtained conc. (ng g^−1^) (LA-ICP-MS)	Expected conc. (ng g^−1^) (ID-ICP-MS)
QC 1	19 ± 3	20 ± 1
QC 2	1420 ± 160	1042 ± 92
QC 3	9128 ± 991	9384 ± 1792

### Bio-imaging of kidney samples: uranium microdistribution

**Fig. 3:**
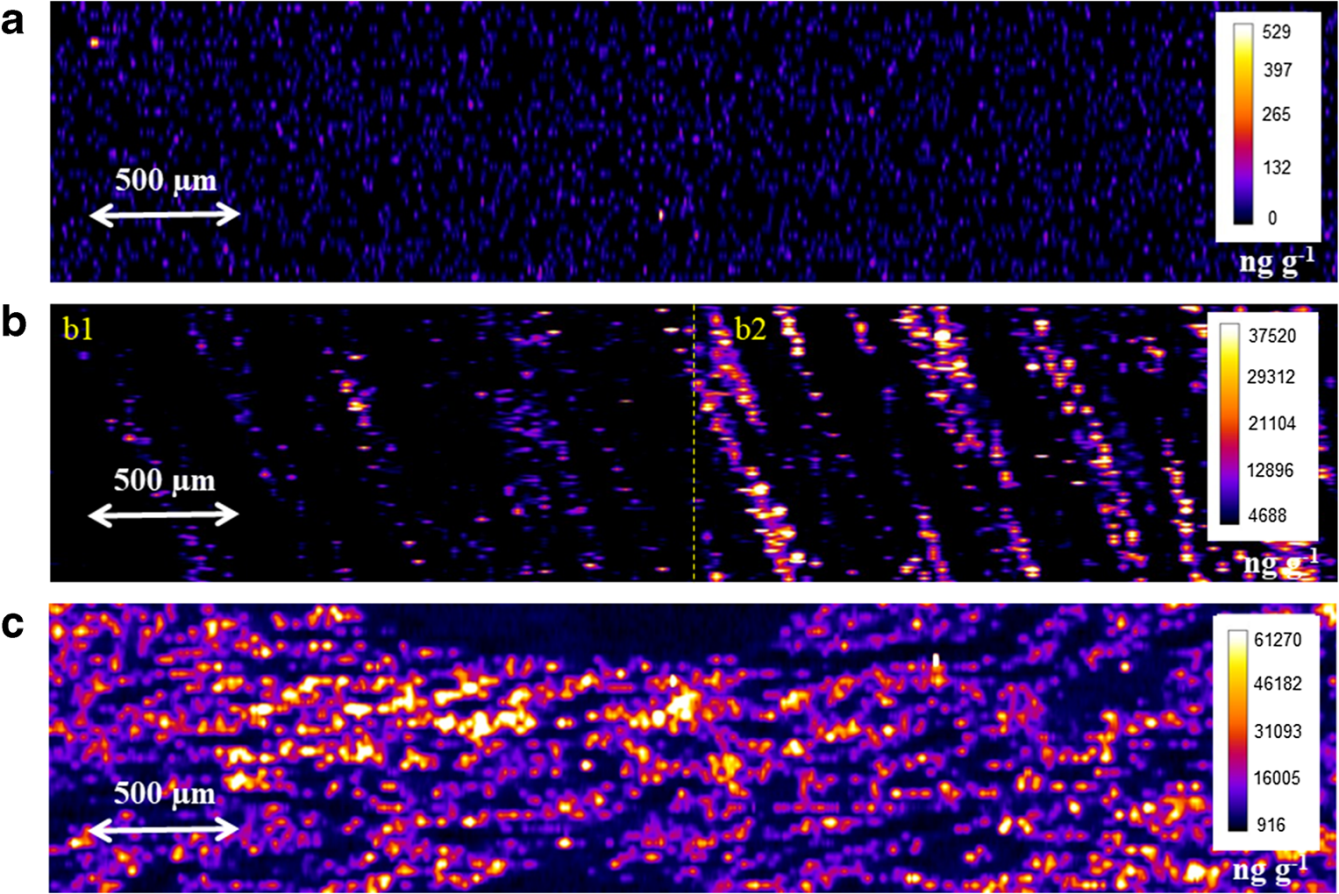
Ablation area in the cortical area of sample 1 (a), medullar (b1) and cortical (b2) areas of sample 2 (b), and cortical area of sample 3 (c). Concentrations are expressed in ng g^−1^

In a first analysis, an area of 2 mm × 8 mm was ablated in the cortical and/or medullar area of sample 1 (whole organ U concentration 4 ng g^−1^), sample 2 (whole organ U concentration 3300 ng g^−1^), and sample 3 (whole organ U concentration 6700 ng g^−1^) in order to verify the accuracy of the developed quantification method (Fig. 3). The average uranium concentrations in the ablated zones are 12 ng g^−1^, 4118 ng g^−1^, and 11,700 ng g^−1^, respectively (Fig. 3a, b, and c). It must be taken into account that the ablated zones represent a small volume of the whole organ which means that the quantification performed by LA-ICP-MS can differ from the quantification performed on whole organ by ICP-MS analysis after acid digestion. With the aim of validating the obtained results, subsequent nine histological cuts (16-μm thickness) of sample 1 (control sample) were taken just immediately after the histological cut reserved for LA-ICP-MS. They were acid digested and analyzed in triplicate by ID-ICP-MS (*n* = 27), resulting in uranium average concentration of 13.5 ± 5.8 ng g^−1^ in agreement with the uranium concentration found by LA-ICP-MS (12 ng g^−1^).

As expected, a high difference in U concentration is observed between the medullar (Fig. 3 b1) and cortical (Fig. 3 b2) area in sample 2 (Fig. 3b). Even if the average uranium concentration in the ablated zone (4118 ng g^−1^) is not far from the ICP-MS concentration in the whole organ (3300 ng g^−1^), the average cortical concentration (5966 ng g^−1^) is up to twofold higher than the average medullary concentration (2452 ng g^−1^). “Hot spots” of U with concentrations ranging between 21,104 and 37,520 ng g^−1^ can be also observed, preferentially located in the cortical area, which means that, in this histological cut, the uranium concentration is up to 11-fold higher compared with the whole organ concentration. These results are in agreement with those published by Homma-Takeda et al. [16]. Similarly, in sample 3, the average uranium concentration in the ablated zone is 11,700 ng g^−1^ (Fig. 3c), which is in accordance with the whole organ concentration. However, very highly concentrated zones are identified, in which the uranium concentration reaches up to 60,000 ng g^−1^, that is up to 10-fold higher than the whole organ uranium concentration (6700 ng g^−1^). Table 4 summarizes the samples used in this study and the obtained results after LA-ICP-MS analysis.

**Table 4:** Samples used for the study (type of sample, ablation zone) and the comparison of obtained results by LA-ICP-MS with those obtained by whole organ ICP-MS analysis after acid digestion

Sample	Type of sample	Ablation zone	Whole organ U average conc. (ICP-MS, ng g^−^1)	U conc. per zone (LA-ICP-MS, ng g^−^1)
1	Control (no U contamination)	Cortex	4^a^	12
2	U contaminated	Cortex/medulla	3256	4118^b^
3	U contaminated	Cortex	6729	11,700

^a^The average uranium concentration per 16-μm thickness histological cut is 13.5 ± 5.8 ng g^−1^, calculated by ID-ICP-MS after acid digestion (*n* = 27). ^b^Cortical concentration, 5966 ng g^−1^; medullary concentration, 2452 ng g^−1^

**Fig. 4:**
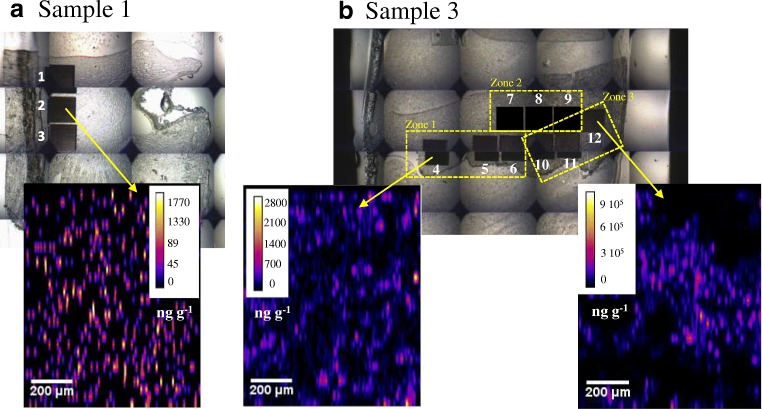
Microscope images of non-contaminated (a) and contaminated (b) kidney histological cuts. Ablation zones are visible both in the medullary and cortical areas

In order to take a step forward, quantification and localization of uranium in kidney tissue, several areas (0.5 × 0.5 mm^2^) were ablated both in the medullar and cortical areas of non-contaminated (sample 1) and the most contaminated (sample 3) samples. Three areas were ablated in the medullary area of the non-contaminated sample (Fig. 4a—1, 2, and 3) from the inner part of the kidney to the near cortical area. The uranium average concentration is 14 ± 5 ng g^−1^. A trend of increasing concentration towards the cortical area is observed, from 11 ng g^−1^ in the inner part of the medullary area to 21 ng g^−1^ near the cortex. Similarly, nine ablations were done in the contaminated sample throughout the entire histological cut: 3 ablations in the medullary area (Fig. 4b—zone 1), 3 ablations in the medulla-cortex intermediate area (Fig. 4b—zone 2), and 3 ablations in the cortical area (Fig. 4b—zone 3). The uranium average concentrations corresponding to these zones are 320 ± 150 ng g^−1^, 1110 ± 715 ng g^−1^, and 38,180 ± 18,550 ng g^−1^, respectively. Table 5 summarizes the concentrations found by LA-ICP-MS in different ablation zones of non-contaminated (sample 1) and most contaminated (sample 3) samples. These results show up the ascendant concentration of uranium from the medulla to cortex by a factor of 120, which is in agreement with the quantitative results obtained by high-energy synchrotron radiation X-ray fluorescence (SR-XRF) analysis published by Homma-Takeda [16]. Additionally, the fact that the uranium accumulates at greater concentrations in the renal cortex is also corroborated by a previous study carried out by Tessier et al. employing secondary ion mass spectrometry (SIMS) microscopy [61].

**Table 5:** Average uranium concentration (ng g^−1^) for each ablation area and per zone. Areas 1–3 correspond to the non-contaminated sample (sample 1) and areas 4–12 correspond to the most contaminated sample

Area	Average conc. U (ng g^−1^) per ablated area	Average conc. U (ng g^−1^) per zone
1	11	14 ± 5
2	12	
3	21	
4	143	320 ± 150
5	386	
6	424	
7	584	1110 ± 715
8	825	
9	1926	
10	18,321	38,180 ± 18,550
11	53,840	
12	45,382	

## Discussion

In this work, the feasibility of an internal standard spiked–gelatine was assessed for its use in quantitative bio-imaging of U in kidney tissue by laser ablation coupled to ICP-MS. Beyond the optimization of the gelatine itself, matrix-matched standards have been also developed. The gelatine was prepared and deposited on the glass slides the day before the tissue cryo-cutting and the LA-ICP-MS analysis was performed in the following days to ensure the most appropriate analytical conditions. This new methodology (U-spiked matrix-matching standards, Tm-spiked gelatine as IS) confirmed the uranium heterogeneous distribution in the kidney with a higher concentration in the cortical area than in the medullar area and allowed its accurate quantification in the analyzed kidney tissue section. Thulium-spiked gel was demonstrated to be adequate for compensation of instrumental drifts during measuring time and matrix effects, which improves the quantification of elemental distributions in biological tissue. The proposed analytical bio-imaging approach was successfully applied for uranium quantification in rat kidneys. The comparison of the calculated average concentration obtained by LA-ICP-MS and the results obtained after liquid ICP-MS analysis were in good accordance. Quantitative images of cryo-sections revealed heterogeneous distribution of uranium within the renal tissue, with cortical uranium concentration up to 120-fold higher than medullary uranium concentration, which is in great accordance with that of the studies found in the literature. To the author’s knowledge, this is the first quantitative study using an internal standard other than C^13^ to precisely quantify and localize the uranium in kidney by LA-ICP-MS. Therefore, this work is an important contribution to study the renal uranium accumulation which, undoubtedly, will help to gain more in-depth knowledge about the mechanisms of uranium deposition in an organism. The future goal is focused on enhancing the image quality by optimizing ablation parameters [62] and the analysis of larger areas for a more accurate cartography of the renal distribution of uranium in the whole organ. Additionally, replacement of kidney matrix-matched standards, which is a time-consuming process and their homogeneity debatable, by spiked gelatine droplets will be considered because of its better homogeneity, its ease of handling, and the no-need of animals. By this way, a better understanding of uranium nephrotoxicity and its implication in the development of kidney cancer in nuclear workers is expected.

## References

[1] Priest ND (2001). Toxicity of depleted uranium. Lancet.

[2] Gueguen Y, Rouas C (2012). Données nouvelles sur la néphrotoxicité de l’uranium. Radioprotection.

[3] Haley DP, Bulger RE, Dobyan DC (1982). The long-term effects of uranyl nitrate on the structure and function of the rat kidney. Virchows Arch B Cell Pathol Incl Mol Pathol.

[4] Vicente-Vicente L, Quiros Y, Pérez-Barriocanal F, López-Novoa JM, López-Hernández FJ, Morales AI (2010). Nephrotoxicity of uranium: pathophysiological, diagnostic and therapeutic perspectives. Toxicol Sci.

[5] Keith S, Faroon O, Roney N, Scinicariello F, Wilbur S, Ingerman L, Llados F, Plewak D, Wohlers D, Diamond G (2013). Toxicological profile for uranium. 3. Health effects.

[6] Homma-Takeda S, Terada Y, Nakata A, Sahoo SK, Yoshida S, Ueno S, Inoue M, Iso H, Ishikawa T, Konishi T, Imaseki H, Shimada Y (2009). Elemental imaging of kidneys of adult rats exposed to uranium acetate. Nucl Instrum Methods B.

[7] Homma-Takeda S, Kokubo T, Terada Y, Suzuki K, Ueno S, Hayao T, Inoue T, Kitahara K, Blyth BJ, Nishimura M, Shimada Y (2013). Uranium dynamics and developmental sensitivity in rat kidney. J Appl Toxicol.

[8] Konishi T, Kodaira S, Itakura Y, Ohsawa D, Homma-Takeda S. Imaging uranium distribution on rat kidney sections through detection of alpha tracks using CR-39 plastic nuclear track detector. Radiat Prot Dosim. 2018. 10.1093/rpd/ncy224 30521045

[9] Stammler L, Uhl A, Mayer B, Keller F (2016). Renal effects and carcinogenicity of occupational exposure to uranium: a meta-analysis. Nephron Extra.

[10] Qu SG, Gao J, Tang B, Yu B, Shen YP, Tu Y. Low-dose ionizing radiation increases the mortality risk of solid cancers in nuclear industry workers: a meta-analysis. Mol Clin Oncol. 2018;8(5). 10.3892/mco.2018.1590 PMC5920205 29725540

[11] Golden AP, Ellis ED, Cohen SS, Mumma MT, Leggett RW, Wallace PW, et al. Updated mortality analysis of the Mallinckrodt uranium processing workers, 1942–2012. Int J Radiat Biol. 2019:1–21. 10.1080/09553002.2019.1569773 30652958

[12] ICRP, 2007. The 2007 recommendations of the Internal Commission on Radiological Protection. ICRP Ann Publication 103. 10.1016/j.icrp.2007.10.003 18082557

[13] Paquet F, Harrison J (2018). ICRP Task Group 95: internal dose coefficients. Ann ICRP.

[14] Paquet F, Houpert P, Blanchardon E, Delissen O, Maubert C, Dhieux B, Moreels AM, Frelon S, Gourmelon P (2006). Accumulation and distribution of uranium in rats after chronic exposure by ingestion. Health Phys.

[15] ICPR, 2017. Occupational intakes of radionuclides: Part 3. ICRP Publication 137. Ann ICRP 46 (3/4): 410–411. 10.1177/0146645317734963 29380630

[16] Homma-Takeda S, Kitahara K, Suzuki K, Blyth BJ, Suya N, Konishi T, Terada Y, Shimada Y (2015). Cellular localization of uranium in the renal proximal tubules during acute renal uranium toxicity. J Appl Toxicol.

[17] Kitahara K, Numako C, Terada Y, Nitta K, Shimada Y, Homma-Takeda S. Uranium XAFS analysis of kidney from rats exposed to uranium. J Synchroton Radiat. 2017;24(2). 10.1107/S1600577517001850 PMC5330292 28244440

[18] Grijalba N, Legrand A, Holler V, Bouvier-Capely C. Renal localization and quantification of uranium in rodent exposed to uranyl nitrate by LA-ICP-MS. 15th International Conference on Laser Ablation. 2019(Book of abstracts):p.xxviii (302).

[19] Jim V, LaViolette C, Briehl M, Ingram J (2017). Spatial distribution of uranium in mice kidneys detected by laser ablation inductively coupled plasma mass spectrometry. J Appl Bioanal.

[20] Russo RE, Mao X, Liu H, Gonzalez J, Mao SS (2002). Laser ablation in analytical chemistry—a review. Talanta..

[21] Sussulini A, Becker JS, Becker JS (2017). Laser ablation ICP-MS: application in biomedical research. Mass Spectrom Rev.

[22] Miliszkiewicz N, Walas S, Tobiasz A (2015). Current approaches to calibration of LA-ICP-MS analysis. J Anal Atom Spectrom.

[23] Pozebon D, Scheffler GL, Dressler VL (2017). Recent applications of laser ablation inductively coupled plasma mass spectrometry (LA-ICP-MS) for biological sample analysis: a follow-up review. J Anal Atom Spectrom.

[24] Hare D, Austin C, Doble P (2012). Quantification strategies for elemental imaging of biological samples using laser ablation-inductively coupled plasma-mass spectrometry. Analyst..

[25] Longerich HP, Jackson SE, Günther D (1996). Inter-laboratory note. Laser ablation inductively coupled plasma mass spectrometric transient signal data acquisition and analyte concentration calculation. J Anal Atom Spectrom..

[26] Limbeck A, Galler P, Bonta M, Bauer G, Nischkauer W, Vanhaecke F (2015). Recent advances in quantitative LA-ICP-MS analysis: challenges and solutions in the life sciences and environmental chemistry. Anal Bioanal Chem.

[27] Becker JS, Zoriy MV, Pickhardt C, Palomero-Gallagher N, Zilles K (2005). Imaging of copper, zinc, and other elements in thin section of human brain samples (hippocampus) by laser ablation inductively coupled plasma mass spectrometry. Anal Chem.

[28] Fitzpatrick AJ, Kurtis Kyser T, Chipley D, Beauchemin D (2008). Fabrication of solid calibration standards by a sol–gel process and use in laser ablation ICPMS. J Anal Atom Spectrom.

[29] Austin C, Hare D, Rawling T, McDonagh AM, Doble P (2010). Quantification method for elemental bio-imaging by LA-ICP-MS using metal spiked PMMA films. J Anal Atom Spectrom..

[30] Kumtabtim U, Siripinyanond A, Auray-Blais C, Ntwari A, Becker JS (2011). Analysis of trace metals in single droplet of urine by laser ablation inductively coupled plasma mass spectrometry. Int J Mass Spectrom.

[31] Bonta M, Hegedus B, Limbeck A (2016). Application of dried-droplets deposited on pre-cut filter paper disks for quantitative LA-ICP-MS imaging of biologically relevant minor and trace elements in tissue samples. Anal Chim Acta.

[32] Villaseñor Á, Boccongelli M, Todolí JL (2018). Quantitative elemental analysis of polymers through laser ablation – inductively coupled plasma by using a dried droplet calibration approach, DDCA. J Anal Atom Spectrom.

[33] Resano M, Belarra MA, García-Ruiz E, Aramendía M, Rello L (2018). Dried matrix spots and clinical elemental analysis. Current status, difficulties, and opportunities. TrAC..

[34] Kröger S, Sperling M, Karst U (2019). Quantitative dried blood spot analysis for metallodrugs by laser ablation-inductively coupled plasma-mass spectrometry. J Trace Elem Med Biol.

[35] Van Malderen SJM, Vergucht E, De Rijcke M, Janssen C, Vincze L, Vanhaecke F (2016). Quantitative determination and subcellular imaging of cu in single cells via laser ablation-ICP-mass spectrometry using high-density microarray gelatin standards. Anal Chem.

[36] Costas-Rodríguez M, Van Acker T, Hastuti AAMB, Devisscher L, Van Campenhout S, Van Vlierberghe H, Vanhaecke F (2017). Laser ablation-inductively coupled plasma-mass spectrometry for quantitative mapping of the copper distribution in liver tissue sections from mice with liver disease induced by common bile duct ligation. J Anal Atom Spectrom..

[37] Šala M, Šelih VS, van Elteren JT (2017). Gelatin gels as multi-element calibration standards in LA-ICP-MS bioimaging: fabrication of homogeneous standards and microhomogeneity testing. Analyst..

[38] Lohöfer F, Hoffmann L, Buchholz R, Huber K, Glinzer A, Kosanke K, Feuchtinger A, Aichler M, Feuerecker B, Kaissis G, Rummeny EJ, Höltke C, Faber C, Schilling F, Botnar RM, Walch AK, Karst U, Wildgruber M. Molecular imaging of myocardial infarction with gadofluorine P – a combined magnetic resonance and mass spectrometry imaging approach. Heliyon. 2018;4(4):e00606. 10.1016/j.heliyon.2018.e00606 PMC5968177 29862367

[39] Van Acker T, Buckle T, Van Malderen SJM, van Willigen DM, van Unen V, van Leeuwen FWB, Vanhaecke F (2019). High-resolution imaging and single-cell analysis via laser ablation-inductively coupled plasma-mass spectrometry for the determination of membranous receptor expression levels in breast cancer cell lines using receptor-specific hybrid tracers. Anal Chim Acta.

[40] Cruz-Alonso M, Fernandez B, Navarro A, Junceda S, Astudillo A, Pereiro R (2019). Laser ablation ICP-MS for simultaneous quantitative imaging of iron and ferroportin in hippocampus of human brain tissues with Alzheimer’s disease. Talanta..

[41] Lohöfer F, Buchholz R, Glinzer A, Huber K, Haas H, Kaissis G, Feuchtinger A, Aichler M, Sporns PB, Höltke C, Stölting M, Schilling F, Botnar RM, Kimm MA, Faber C, Walch AK, Zernecke A, Karst U, Wildgruber M (2020). Mass spectrometry imaging of atherosclerosis-affine gadofluorine following magnetic resonance imaging. Sci Rep-UK.

[42] Thompson M, Chenery S, Brett L (1989). Calibration studies in laser ablation microprobe-inductively coupled plasma atomic emission spectrometry. J Anal Atom Spectrom.

[43] Barats A, Pecheyran C, Amouroux D, Dubascoux S, Chauvaud L, Donard OF (2007). Matrix-matched quantitative analysis of trace-elements in calcium carbonate shells by laser-ablation ICP-MS: application to the determination of daily scale profiles in scallop shell (Pecten maximus). Anal Bioanal Chem.

[44] Chirinos J, Oropeza D, González J, Zorba V, Russo RE (2016). Analysis of plant leaves using laser ablation inductively coupled plasma optical emission spectrometry: use of carbon to compensate for matrix effects. Appl Spectrosc.

[45] Hanć A, Małecka A, Kutrowska A, Bagniewska-Zadworna A, Tomaszewska B, Barałkiewicz D (2016). Direct analysis of elemental biodistribution in pea seedlings by LA-ICP-MS, EDX and confocal microscopy: imaging and quantification. Microchem J.

[46] Konz I, Fernández B, Fernández ML, Pereiro R, González H, Álvarez L, Coca-Prados M, Sanz-Medel A (2013). Gold internal standard correction for elemental imaging of soft tissue sections by LA-ICP-MS: element distribution in eye microstructures. Anal Bioanal Chem.

[47] Konz I, Fernández B, Fernández ML, Pereiro R, González-Iglesias H, Coca-Prados M, Sanz-Medel A (2014). Quantitative bioimaging of trace elements in the human lens by LA-ICP-MS. Anal Bioanal Chem.

[48] Moraleja I, Esteban-Fernandez D, Lazaro A, Humanes B, Neumann B, Tejedor A, Luz Mena M, Jakubowski N, Gómez-Gómez MM (2016). Printing metal-spiked inks for LA-ICP-MS bioiimaging internal standardization: comparison of the different nephrotoxic behavior of cisplatin, carboplatin, and oxaliplatin. Anal Bioanal Chem.

[49] Hoesl S, Neumann B, Techritz S, Sauter G, Simon R, Schlüter H, Linscheid MW, Theuring F, Jakubowski N, Mueller L (2016). Internal standardization of LA-ICP-MS immuno imaging via printing of universal metal spiked inks onto tissue sections. J Anal Atom Spectrom..

[50] Moraleja I, Mena ML, Lazaro A, Neumann B, Tejedor A, Jakubowski N, Gómez-Gómez MM, Esteban-Fernández D (2018). An approach for quantification of platinum distribution in tissues by LA-ICP-MS imaging using isotope dilution analysis. Talanta..

[51] Neumann B, Hosl S, Schwab K, Theuring F, Jakubowski N (2020). Multiplex LA-ICP-MS bio-imaging of brain tissue of a parkinsonian mouse model stained with metal-coded affinity-tagged antibodies and coated with indium-spiked commercial inks as internal standards. J Neurosci Methods.

[52] Austin C, Fryer F, Lear J, Bishop D, Hare D, Rawling T, Kirkup L, McDonald A, Doble P (2011). Factors affecting internal standard selection for quantitative elemental bio-imaging of soft tissues by LA-ICP-MS. J Anal Atom Spectrom..

[53] Frick DA, Günther D (2012). Fundamental studies on the ablation behaviour of carbon in LA-ICP-MS with respect to the suitability as internal standard. J Anal Atom Spectrom..

[54] Becker JS, Matusch A, Wu B (2014). Bioimaging mass spectrometry of trace elements – recent advance and applications of LA-ICP-MS: a review. Anal Chim Acta.

[55] Poisson C, Stefani J, Manens L, Delissen O, Suhard D, Tessier C, Dublineau I, Guéguen Y (2014). Chronic uranium exposure dose-dependently induces glutathione in rats without any nephrotoxicity. Free Radic Res.

[56] Qin Z, Caruso JA, Lai B, Matusch A, Becker JS (2011). Trace metal imaging with high spatial resolution: applications in biomedicine. Metallomics..

[57] Hare DJ, George JL, Bray L, Volitakis I, Vais A, Ryan TM, Cherny RA, Bush AI, Masters CL, Adlard PA, Doblet PA, Finkelstein DI (2014). The effect of paraformaldehyde fixation and sucrose cryoprotection on metal concentration in murine neurological tissue. J Anal Atom Spectrom..

[58] Bonta M, Török S, Hegedus B, Döme B, Limbeck A (2017). A comparison of sample preparation strategies for biological tissues and subsequent trace element analysis using LA-ICP-MS. Anal Bioanal Chem.

[59] Suhard D, Tessier C, Manens L, Rebière F, Tack K, Agarande M, Gueguen Y (2018). Intracellular uranium distribution: comparison of cryogenic fixation versus chemical fixation methods for SIMS analysis. Microsc Res Tech.

[60] Bioanalytical method validation: guidance for industry, (2018).

[61] Tessier C, Suhard D, Rebière F, Souidi M, Dublineau I, Agarande M (2012). Uranium microdistribution in renal cortex of rats after chronic exposure: a study by secondary ion mass spectrometry microscopy. Microsc Microanal.

[62] van Elteren JT, Šelih VS, Šala M (2019). Insights into the selection of 2D LA-ICP-MS (multi)elemental mapping conditions. J Anal Atom Spectrom..

